# Normalization of hepatic ChREBP activity does not protect against liver disease progression in a mouse model for Glycogen Storage Disease type Ia

**DOI:** 10.1186/s40170-023-00305-3

**Published:** 2023-04-21

**Authors:** Martijn G. S. Rutten, Yu Lei, Joanne H. Hoogerland, Vincent W. Bloks, Hong Yang, Trijnie Bos, Kishore A. Krishnamurthy, Aycha Bleeker, Mirjam H. Koster, Rachel E. Thomas, Justina C. Wolters, Hilda van den Bos, Gilles Mithieux, Fabienne Rajas, Adil Mardinoglu, Diana C. J. Spierings, Alain de Bruin, Bart van de Sluis, Maaike H. Oosterveer

**Affiliations:** 1 Department of Pediatrics, University of Groningen, University Medical Center Groningen, Groningen, The Netherlands; 2 Science for Life Laboratory, KTH - Royal Institute of Technology, Stockholm, Sweden; 3 Department of Laboratory Medicine, University of Groningen, University Medical Center Groningen, Groningen, The Netherlands; 4 Department of Biomolecular Health Sciences, Faculty of Veterinary Medicine, Utrecht University, Utrecht, The Netherlands; 5 European Research Institute for the Biology of Ageing (ERIBA), University of Groningen, University Medical Center Groningen, Groningen, The Netherlands; 6 Institut National de La Santé Et de La Recherche Médicale, U1213 Lyon, France; 7 Université de Lyon, Lyon, France; 8 Université Lyon 1, Villeurbanne, France

**Keywords:** Glycogen Storage Disease type 1a, Carbohydrate Response Element Binding Protein, Hepatomegaly, Yes Associated Protein, Cyclic GMP-AMP synthase-stimulator of interferon genes (cGAS-STING)

## Abstract

**Background:**

Glycogen storage disease type 1a (GSD Ia) is an inborn error of metabolism caused by a defect in glucose-6-phosphatase (G6PC1) activity, which induces severe hepatomegaly and increases the risk for liver cancer. Hepatic GSD Ia is characterized by constitutive activation of Carbohydrate Response Element Binding Protein (ChREBP), a glucose-sensitive transcription factor. Previously, we showed that ChREBP activation limits non-alcoholic fatty liver disease (NAFLD) in hepatic GSD Ia. As ChREBP has been proposed as a pro-oncogenic molecular switch that supports tumour progression, we hypothesized that ChREBP normalization protects against liver disease progression in hepatic GSD Ia.

**Methods:**

Hepatocyte-specific *G6pc* knockout (L-*G6pc*^−/−^) mice were treated with AAV-shChREBP to normalize hepatic ChREBP activity.

**Results:**

Hepatic ChREBP normalization in GSD Ia mice induced dysplastic liver growth, massively increased hepatocyte size, and was associated with increased hepatic inflammation. Furthermore, nuclear levels of the oncoprotein Yes Associated Protein (YAP) were increased and its transcriptional targets were induced in ChREBP-normalized GSD Ia mice. Hepatic ChREBP normalization furthermore induced DNA damage and mitotic activity in GSD Ia mice, while gene signatures of chromosomal instability, the cytosolic DNA-sensing cGAS-STING pathway, senescence, and hepatocyte dedifferentiation emerged.

**Conclusions:**

In conclusion, our findings indicate that ChREBP activity limits hepatomegaly while decelerating liver disease progression and protecting against chromosomal instability in hepatic GSD Ia. These results disqualify ChREBP as a therapeutic target for treatment of liver disease in GSD Ia. In addition, they underline the importance of establishing the context-specific roles of hepatic ChREBP to define its therapeutic potential to prevent or treat advanced liver disease.

**Supplementary Information:**

The online version contains supplementary material available at 10.1186/s40170-023-00305-3.

## Background

Glycogen Storage Disease type Ia (GSD Ia) (MIM#232,200) is a rare inborn error of metabolism (IEM) caused by mutations in the gene encoding for the catalytic subunit of glucose-6-phosphatase (*G6PC1* (*G6pc* in mice), G6Pase-α) [1], which is expressed in liver, kidney, and intestine, where it converts glucose-6-phosphate (G6P) into glucose. Patients primarily display severe metabolic liver disease, characterized by hepatomegaly and non-alcoholic fatty liver disease (NAFLD), while liver tumour development represents the major long-term complication of GSD Ia, affecting up to 70% of patients by the age of 30 years [2].

Carbohydrate Response Element Binding Protein (ChREBP, also known as MLXIPL, MONDOB, or WBSCR14) is the major glucose-sensitive transcription factor in hepatocytes [3], and ChREBP and its regulated pathways are activated in hepatic GSD Ia [4–7]. We previously showed that short-term normalization of ChREBP activity aggravates hepatomegaly and NAFLD in hepatocyte-specific GSD Ia mice [8]. These findings suggest that sustained hepatic ChREBP normalization in hepatic GSD Ia may drive advanced liver disease elements, including hepatic inflammation, liver fibrosis, hepatocellular death and/or oncogenic transformation. On the other hand, evidence that ChREBP-regulated pathways represent a typical hallmark of many cancer cells has accumulated [9]. Consistently, ChREBP has been linked to the incidence and prognosis of hepatocellular carcinoma (HCC) [10–14]. ChREBP-deficient mice are protected against HCC development in an oncogene-specific manner, and ChREBP deficiency inhibits growth of β-catenin/YAP-driven hepatoblastomas [11, 15]. Moreover, reduced ChREBP expression inhibits hepatocellular proliferation through oxidative stress-induced, p53-mediated cell cycle arrest in vitro [16], while ChREBP-deficient hepatocytes show impaired proliferation rates during liver repopulation in vivo [15]. Combined, these studies indicate that ChREBP serves as a competent factor for cell growth and liver tumour progression.

These previous studies from our laboratory and others suggest context-specific roles of hepatic ChREBP in advanced liver disease, in particular hepatocellular tumour susceptibility, which are of critical importance to establish the therapeutic potential of ChREBP for the treatment of liver disease in GSD Ia patients. In the current study we therefore investigated the impact of prolonged ChREBP normalization on liver disease progression in hepatic GSD Ia mice. Our data show that normalization of hepatic ChREBP activity sensitizes liver-specific GSD Ia mice to advanced liver disease development, DNA damage, cellular senescence, as well as hepatocellular proliferation and dedifferentiation, suggesting increased susceptibility for hepatocarcinogenesis.

## Methods

### AAV-shRNA construction and production

See Supplement.

### Animals

Male adult *G6pc-*floxed *Alb*-Cre negative (B6.*G6pc*^lox/lox^) and *G6pc-*floxed *Alb*-Cre positive (B6.*G6pc*^lox/lox^.SA^creERT2/w^ mice) on a C57BL/6 J background were infected with shRNAs directed against ChREBP (AAV-shChREBP) or a scrambled control (AAV-shScramble (shSCR)) (1 × 10^12^ particles per mouse) by intravenous injection into the retro-orbital plexus under isoflurane anaesthesia [8]. At 11–12 days after AAV-shRNA administration, all mice received i.p. injections of tamoxifen for 5 consecutive days to generate liver-specific *G6pc*-deficient mice (L-*G6pc*^−/−^) and wildtype littermates (L-*G6pc*^+/+^). Nonfasted animals were sacrificed for tissue collection at 8AM at 10 or 25–26 days after the last treatment (dpt). Two shChREBP/L-*G6pc*^−/−^ mice were euthanized at 21 dpt because a humane endpoint was reached, yet were included in the analyses. Absolute liver weight of the 10-day follow-up study cohort has previously been reported [8]. For further details, see Supplementary Materials & Methods. All experimental procedures were approved by the Institutional Animal Care and Use Committee of the University of Groningen and are in line with the Guide for the Care and Use of Laboratory Animals.

### Histological and pathological analysis of the liver

See Supplement.

### Biochemical assays

See Supplement.

### Gene expression analysis, RNA-sequencing, gene set enrichment analysis (GSEA) and reporter transcription factors analysis

See Supplement.

### Targeted proteomics, SDS-PAGE, and Western Blot

See Supplement.

### Ploidy analysis

For analysis of hepatocyte ploidy, ~20 mg of frozen powdered liver tissue was used for nuclei isolation (as described [17]) in lysis buffer (10 mM Tris–HCl (pH 8), 0.32 M Sucrose, 5 mM CaCl_2_, 3 mM Mg(Ac)_2_, and 0.1 mM EDTA, with fresh addition of 1 mM DTT and 0.1% Triton X-100). In short, a 50–100 µm filter was placed on a 50 mL Falcon tube, and liver powder was poured onto the filter. Liver powder was stepwise and gently homogenized in 1.5 mL lysis buffer and pushed through the filter using a 5 mL syringe plunger. The resulting 1.5 mL nuclear suspension was transferred to a 1.5 or 2.0 mL tube, and supplemented with ~ 25,000 control cells (diploid human GFP-positive cells). Nuclei were spun down at 500xg for 5 min at 4 °C and the pellet was resuspended in 300–1000 µL PBS/BSA with Hoechst/PI DNA dyes (10 μg/mL for both). Nuclei were filtered through 35 µm FACS tubes and analysed on the Canto FACS machine (BD Biosciences) for ploidy analysis.

### Statistics

Data in figures is presented as dot plots with median ± interquartile range (IQR), unless stated otherwise. Data in tables is presented as median (range), unless stated otherwise. Data in heatmaps represent z-score normalized values. Statistical analysis was performed using BrightStat and GraphPad PRISM software. Differences between multiple groups were tested by a Kruskal Wallis H-test followed by post-hoc Conover pairwise comparisons. *P* values < 0.001 (***, ^^^^^, or ^###^), 0.001 to 0.01 (**, ^^^^, or ^##^), and 0.01 to 0.05 (*, ^^^, or ^#^) were considered significant.

## Results

### Normalization of hepatic ChREBP expression in GSD Ia liver induces oxidative stress, p53 activation, and cell cycle inhibition while inducing mitosis

**Fig. 1:**
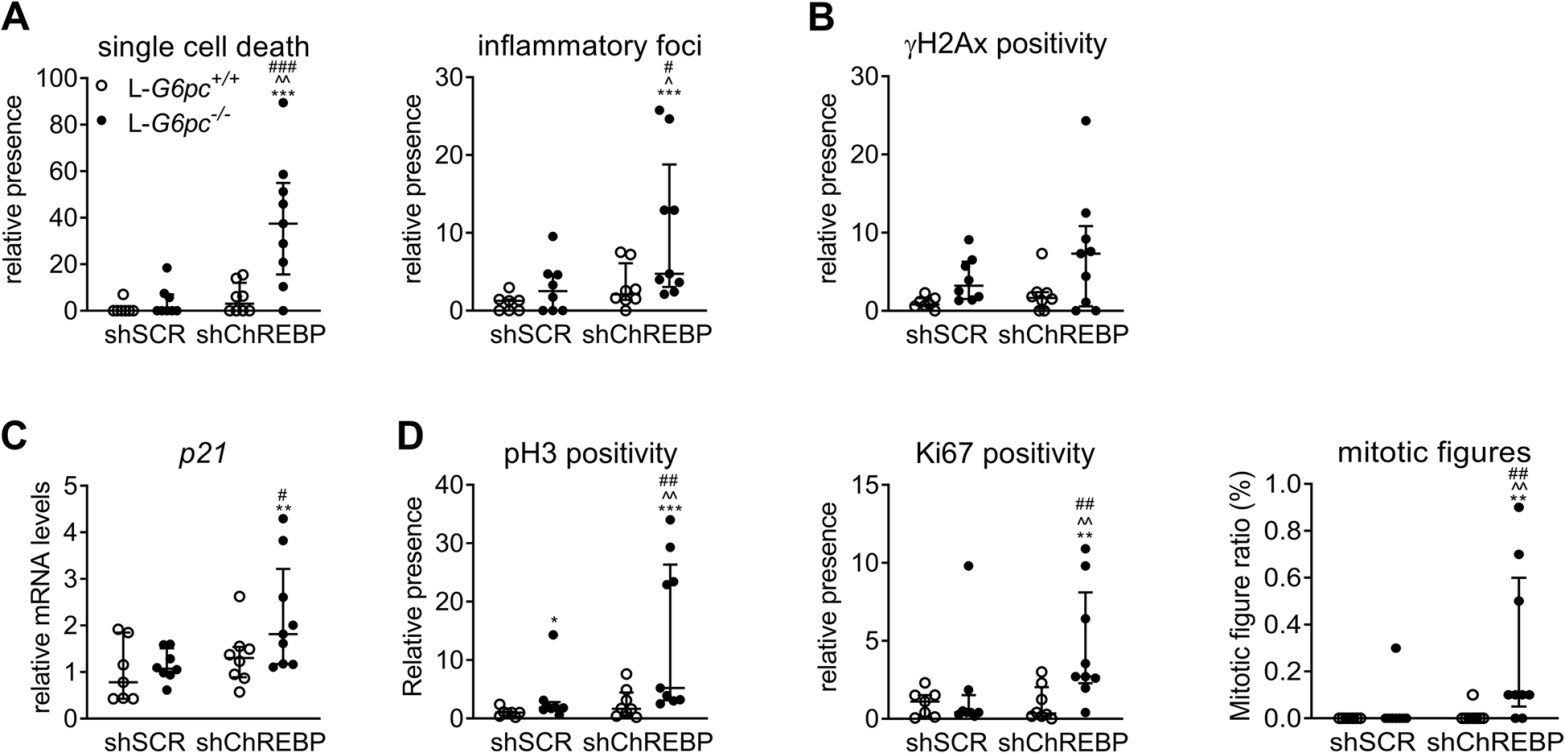
ChREBP knockdown in hepatic GSD Ia mice causes hepatocyte death, inflammation, DNA damage, and proliferation. (**A**) Single cell death and inflammatory foci, (**B**) γH2Ax positivity, (**C**) *p21* (*Cdkn1a*) expression, and (**D**) pH3 and Ki67 positivity and mitotic figures in livers after 10 days of shChREBP/L-*G6pc*^−/−^. A-D: median ± interquartile range; Kruskal Wallis H-test, post-hoc Conover pairwise comparisons, *p < 0.05, **p < 0.01, ***p < 0.001 vs shSCR/L-*G6pc*^+/+^; ^ vs shChREBP/L-*G6pc*^+/+^; # vs shSCR/L-*G6pc*.^−/−^ (*n* = 7–9)

We previously showed that short-term normalization of ChREBP activity aggravates hepatomegaly and NAFLD in a mouse model for hepatic GSD Ia [8]. Here, histopathological analysis revealed an increase in single cell death and inflammatory foci in shChREBP/L-*G6pc*^−/−^ mice, while the number of γH2Ax-positive hepatocytes tended to increase (Fig. 1A-B, S1A). This was paralleled by a significant induction of the p53-target gene *p21* (Fig. 1C). Moreover, the number of pH3- and Ki67-positive hepatocytes and mitotic figures was increased in shChREBP/L-*G6pc*^−/−^ mice (Fig. 1D, S1A). Complementary Gene Set Enrichment Analysis (GSEA) of RNA expression data (Table 1) revealed that normalization of hepatic ChREBP expression in GSD Ia liver induces oxidative stress, apoptosis, p53 activation, cell cycle inhibition, hepatocyte death, chromosomal instability, DNA damage, cyclic GMP-AMP synthase (cGAS)-stimulator of interferon genes (STING) pathway (cGAS-STING) activation, cellular senescence, inflammatory response, epithelial mesenchymal transition, and mitotic activity within a timeframe of two weeks.

**Table 1:** Gene set enrichment analysis on RNA-seq data of short-term shSCR- and shChREBP-treated L-*G6pc*^+*/*+^ and L-*G6pc*^*−/−*^ mice

	L-*G6pc*^*−/−*^ shSCR vs L-*G6pc*^+*/*+^ shSCR			L-*G6pc*^+*/*+^ shChREBP vs L-*G6pc*^+*/*+^ shSCR			L-*G6pc*^*−/−*^ shChREBP vs L-*G6pc*^*−/−*^ shSCR		
Gene set (size^a^)	NES	Nominal p-value	FDR q-value	NES	Nominal p-value	FDR q-value	NES	Nominal p-value	FDR q-value
P53 pathway (158)	1.22	0.109	0.286	**1.54**	**0.000**	**0.034**	**1.53**	**0.002**	**0.026**
G2/M checkpoint (153)	**1.66**	**0.000**	**0.018**	0.91	0.704	0.813	**1.93**	**0.000**	**0.001**
Oxidative stress (25)	-0.97	0.474	1.000	1.12	0.295	0.479	**1.47**	**0.045**	**0.187**
Apoptosis (122)	1.11	0.265	0.416	**1.54**	**0.005**	**0.032**	**1.66**	**0.000**	**0.012**
CIN29 (15)	**2.15**	**0.000**	**0.000**	**1.97**	**0.002**	**0.002**	**2.07**	**0.000**	**0.001**
cGAS-STING (39)	-1.10	0.289	0.627	1.03	0.402	0.607	**1.90**	**0.000**	**0.001**
MMC2-senescence (133)	**1.33**	**0.046**	**0.155**	**1.79**	**0.000**	**0.007**	**2.19**	**0.000**	**0.000**
Inflammatory response (114)	1.03	0.408	0.488	**1.94**	**0.000**	**0.002**	**1.98**	**0.000**	**0.001**
Epithelial mesenchymal transition (123)	-1.02	0.408	0.691	**1.81**	**0.000**	**0.007**	**2.01**	**0.000**	**0.001**

^a^size: refers to the size of the gene set after filtering out those genes that were not in the expression data set

### Prolonged hepatic ChREBP normalization in L-*G6pc*^−/−^ mice induces extreme hepatomegaly and sensitizes to hepatic inflammation

**Fig. 2:**
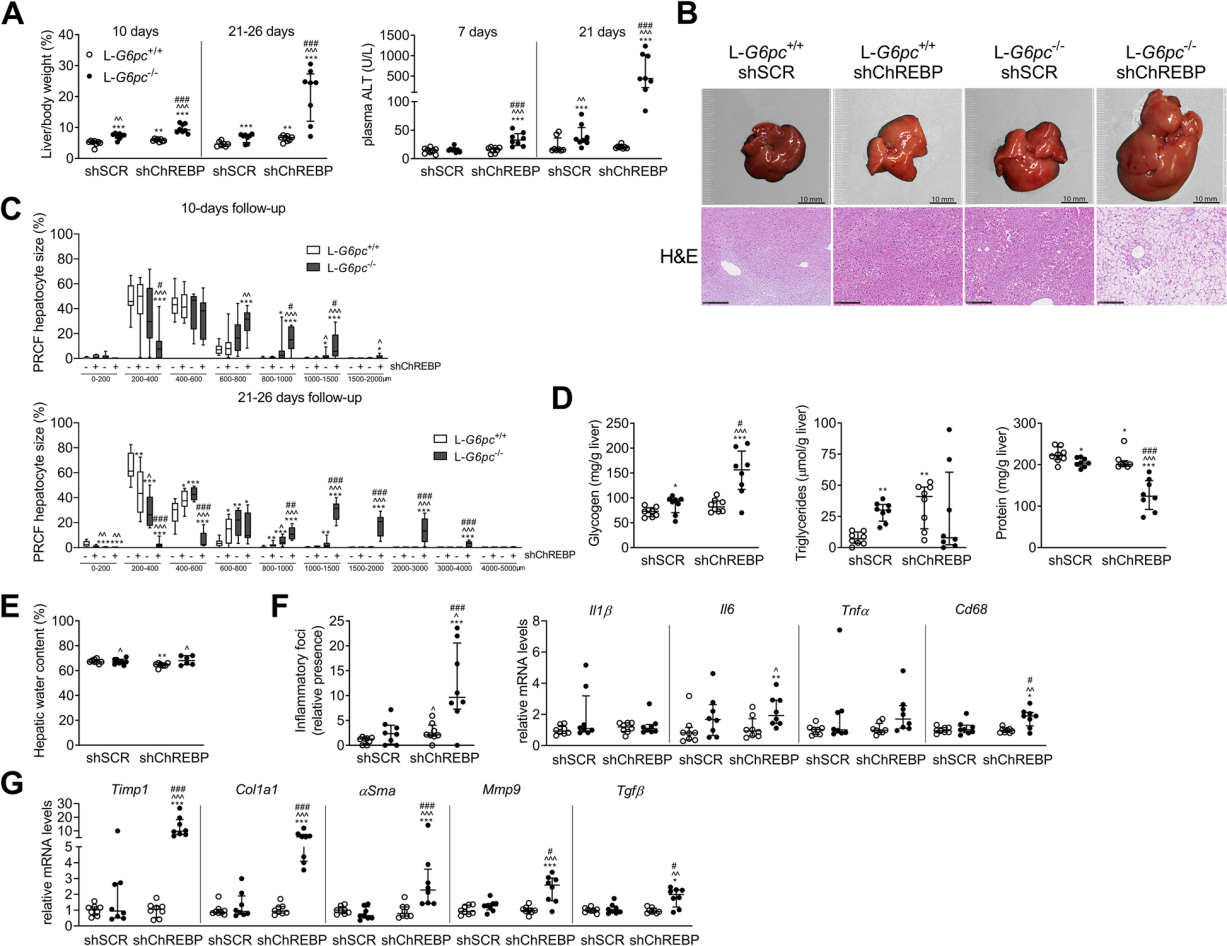
Prolonged hepatic ChREBP normalization in L-*G6pc*^−/−^ mice progressively induces hepatomegaly and sensitizes to hepatic inflammation. (**A**) Liver weight and plasma ALT levels in shChREBP/L-*G6pc*^−/−^ mice (*n* = 8). (**B**) Representative macroscopic liver photos and photos of H&E stainings of livers, and (**C**) Percent relative cumulative frequency (PRCF) of hepatocyte size. (**D**) Hepatic glycogen, triglyceride, and protein content (*n* = 8), (**E**) hepatic water content, (**F**) number of inflammatory foci and inflammatory gene expression, and (**G**) fibrosis marker gene expression in livers of shChREBP/L-*G6pc*^−/−^ mice (*n* = 6–9). A, D-G: median ± interquartile range. C: box-and-whisker plots. A-G: Kruskal Wallis H-test, post-hoc Conover pairwise comparisons, *p < 0.05, **p < 0.01, ***p < 0.001 vs shSCR/L-*G6pc*^+/+^; ^ vs shChREBP/L-*G6pc*^+/+^; # vs shSCR/L-*G6pc*^−/−^

As these early changes suggested acceleration of metabolic-associated fatty liver disease towards advanced liver disease in response to shChREBP, we next evaluated the hepatic effects of prolonged normalized ChREBP activity in L-*G6pc*^−/−^ mice (Fig. S2A). Three weeks of ChREBP normalization reduced fed blood glucose levels and increased plasma ketone bodies, while body weight remained unaffected (Table 2). It furthermore exacerbated hepatomegaly as compared to 10-day ChREBP normalization (Fig. 2A-B, Table 2), and caused a concomitant progressive increase in plasma ALT levels (Fig. 2A) and hepatocyte vacuolization (Fig. 2B), highlighting the progressive nature of the liver disease. Hepatic G6P and glycogen contents were further increased in shChREBP/L-*G6pc*^−/−^ mice, while hepatic triglyceride contents varied, and relative hepatic protein content was reduced (Fig. 2D, Table 2). Hepatic water content was not different between shSCR/L-*G6pc*^−/−^ and shChREBP/L-*G6pc*^−/−^ mice (Fig. 2E). Livers of shChREBP/L-*G6pc*^−/−^ mice showed a marked increase in inflammatory foci, in line with GSEA data on short-term ChREBP normalization in hepatic GSD Ia (Table 1), while the expression of inflammatory genes *Il1β*, *Il6*, *Tnfα*, and *Cd68* was minimally or not (significantly) induced (Fig. 2F). The expression of fibrosis marker genes was increased in shChREBP/L-*G6pc*^−/−^ livers (Fig. 2G). Yet, at the histological level, this was not paralleled by enhanced hepatic collagen deposition (data not shown). These data indicate that prolonged hepatic ChREBP normalization in GSD Ia progressively exacerbates hepatomegaly and hepatocyte hypertrophy and predisposes to hepatic inflammation.

**Table 2:** General data of prolonged shSCR- and shChREBP-treated L-*G6pc*^+*/*+^ and L-*G6pc*^*−/−*^ mice

Variable Median (range)	L-*G6pc*^+*/*+^ shSCR	L-*G6pc*^+*/*+^ shChREBP	L-*G6pc*^*−/−*^ shSCR	L-*G6pc*^*−/−*^ shChREBP
Body weight (g)	29.3 (27.8–31.6)	27.4 (25.6–29.2)	29.1 (25.3–30.5)	27.6 (21.1–32.8)
Liver weight (g)	1.27 (1.14–1.75)	1.89 (1.28–2.10)^**^	2.06 (1.17–2.39)^***^	5.72 (2.15–9.20)^***^^^###^
Blood glucose (mmol/L)	11.1 (7.7–14.2)	10.4 (8.8–14.2)	9.2 (4.9–13.1)	4.9 (1.1–11.5)^***^^#^
**Liver**				
G6P (µmol/g liver)	0.47 (0.29–0.66)	0.53 (0.31–0.63)	1.38 (0.77–3.04)^***^^^^	1.71 (1.28–3.60)^***^^^^
G6P (µmol/liver)	0.65 (0.41–0.81)	1.00 (0.49–1.19)^*^	1.99 (1.50–6.26)^***^^^^	11.58 (2.75–19.15)^***^^^##^
Glycogen (mg/liver)	98 (68–136)	163 (93–224)^*^	190 (71–234)^**^	915 (151–1926)^***^^^##^
Triglycerides (µmol/liver)	8.7 (0.1–23.9)	79.3 (7.6–106.1)^***^	60.8 (22.3–80.7)^**^	50.3 (0.6–238.9)^**^
Free cholesterol (µmol/g liver)	3.94 (2.70–4.54)	3.36 (3.23–3.91)^*^	3.46 (2.97–4.02)	2.20 (1.13–5.43)^**^
Free cholesterol (µmol/liver)	5.08 (3.74–6.44)	6.31 (4.88–7.01)	6.92 (4.71–8.23)^**^	11.73 (10.23–14.48)^***^^^###^
Cholesteryl-esters (µmol/g liver)	0.68 (0.02–0.94)	1.80 (0.57–3.02)^**^	1.85 (1.16–3.03)^**^	1.23 (0.10–5.47)^*^
Cholesteryl-esters (µmol/liver)	0.83 (0.03–1.65)	3.57 (0.73–4.78)^**^	3.23 (2.26–5.11)^***^	7.73 (0.79–11.77)^***^
Total cholesterol (µmol/g liver)	4.45 (2.91–5.27)	5.13 (3.98–6.51)	5.27 (4.14–6.68)	3.36 (1.33–10.90)
Total cholesterol (µmol/liver)	5.87 (4.02–7.93)	10.01 (5.73–10.73)^**^	9.93 (6.97–13.15)^***^	20.06 (11.03–24.79)^***^^^###^
Protein (mg/liver)	300 (241–401)	381 (290–412)	430 (229–481)^**^	656 (394–821)^***^^^##^
**Plasma**				
Lactate (mmol/L)	3.94 (3.46–5.83)	4.72 (3.53–6.09)	5.62 (4.02–7.17)	5.03 (2.65–6.38)
Total ketone bodies (mmol/L)	0.072 (0.055–0.112)	0.087 (0.055–0.165)	0.082 (0.059–0.197)	0.123 (0.090–0.224)^**^#^
3HB (mmol/L)	0.069 (0.051–0.109)	0.084 (0.052–0.161)	0.079 (0.056–0.192)	0.121 (0.087–0.219)^**^#^
ACA (mmol/L)	0.003 (0.002–0.007)	0.004 (0.003–0.007)	0.003 (0.002–0.005)	0.003 (0.000–0.005)
FFA (µmol/L)	153 (96–259)	166 (129–254)	224 (69–306)	167 (97–409)

*p < 0.05, **p < 0.01, ***p < 0.001 vs shSCR/L-*G6pc*^+/+^; ^ vs shChREBP/L-*G6pc*^+/+^; # vs shSCR/L-*G6pc*^-/-^

### Prolonged hepatic ChREBP normalization in L-*G6pc*^−/−^ mice promotes the transcriptional activity of Yes Associated Protein (YAP)

**Fig. 3:**
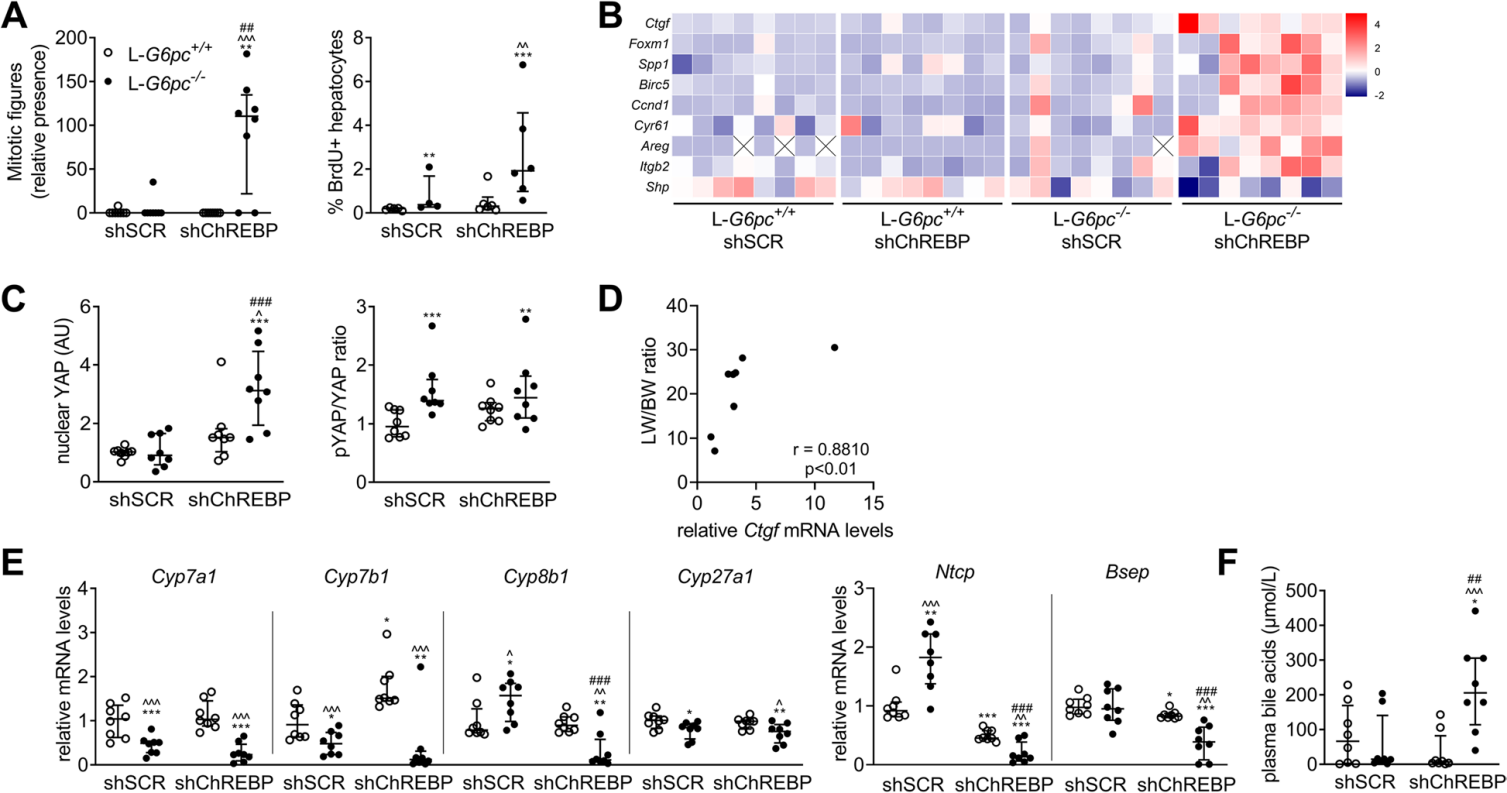
Prolonged hepatic ChREBP normalization in L-*G6pc*^−/−^ mice promotes Yes Associated Protein (YAP) transcriptional activity. Data after 21–26 days of shChREBP/L-*G6pc*^−/−^ and *n* = 8, unless stated otherwise. (**A**) Mitotic figures and BrdU positivity (*n* = 4–6, 20–21 days). (**B**) YAP-target genes and *Shp*. (**C**) YAP nuclear protein and whole liver lysate pYAP/YAP ratio (Blots/Ponceau S: Fig. S2C-E). (**D**) Correlations between liver weight and *Ctgf* expression in shChREBP/L-*G6pc*^−/−^ mice. (**E**) Expression of bile acid synthesis enzymes and transporters, and (**F**) Total plasma bile acid levels. A/C/F-G: median ± interquartile range. E: box-and-whisker plots. A/C/F-G: Kruskal Wallis H-test, post-hoc Conover pairwise comparisons, *p < 0.05, **p < 0.01, ***p < 0.001 vs shSCR/L-*G6pc*^+/+^; ^ vs shChREBP/L-*G6pc*^+/+^; # vs shSCR/L-*G6pc*^−/−^

To further investigate the origin of the extreme liver enlargement observed in hepatic GSD Ia mice upon prolonged ChREBP normalization (Fig. 2A), we next assessed hepatocyte proliferation. Indeed, livers of shChREBP/L-*G6pc*^−/−^ mice showed an increase in mitotic figures and the number of BrdU-positive hepatocytes (Fig. 3A, Fig. S2B). They also exhibited an induction of YAP target genes (Fig. 3B) in parallel to increased nuclear YAP protein levels, while p-YAP/YAP ratios remained unaffected as compared to shSCR-treated controls (Fig. 3C). Interestingly, within shChREBP/L-*G6pc*^−/−^ mice, relative liver weights, mRNA levels of YAP-target gene *Ctgf* (*Ccn2*), and hepatic glycogen contents were positively correlated (Fig. 3D, S2F). In line with our previous work [18], ChREBP normalization in L-*G6pc*^−/−^ mice suppressed the expression of *Cyp8b1* (Fig. 3E). It furthermore reduced the hepatic expression of bile acid transporters *Ntcp* (*Slc10a1*) and *Bsep* (*Abcb11*) (Fig. 3E) while increasing plasma bile acid levels (Fig. 3F). Interestingly, relative liver weight correlated significantly yet moderately with total plasma bile acid (*r* = 0.3000, *p* < 0.05) across different study cohorts (*n* = 64). In the current study cohort, plasma bile acids levels also positively correlated with *Ctgf* mRNA levels (*r* = 0.6884, *p* < 0.0001 (*n* = 32)). Combined, these data indicate that prolonged hepatic ChREBP normalization in hepatic GSD Ia mice enhances YAP activity, which may be mediated by hepatocyte-autonomous effects, such as cellular glycogen accumulation, and/or by hepatic bile acid sensing.

### Prolonged ChREBP knockdown in L-*G6pc*^−/−^ mice induces hepatocyte DNA damage, cellular senescence, and hepatocyte dedifferentiation

**Fig. 4:**
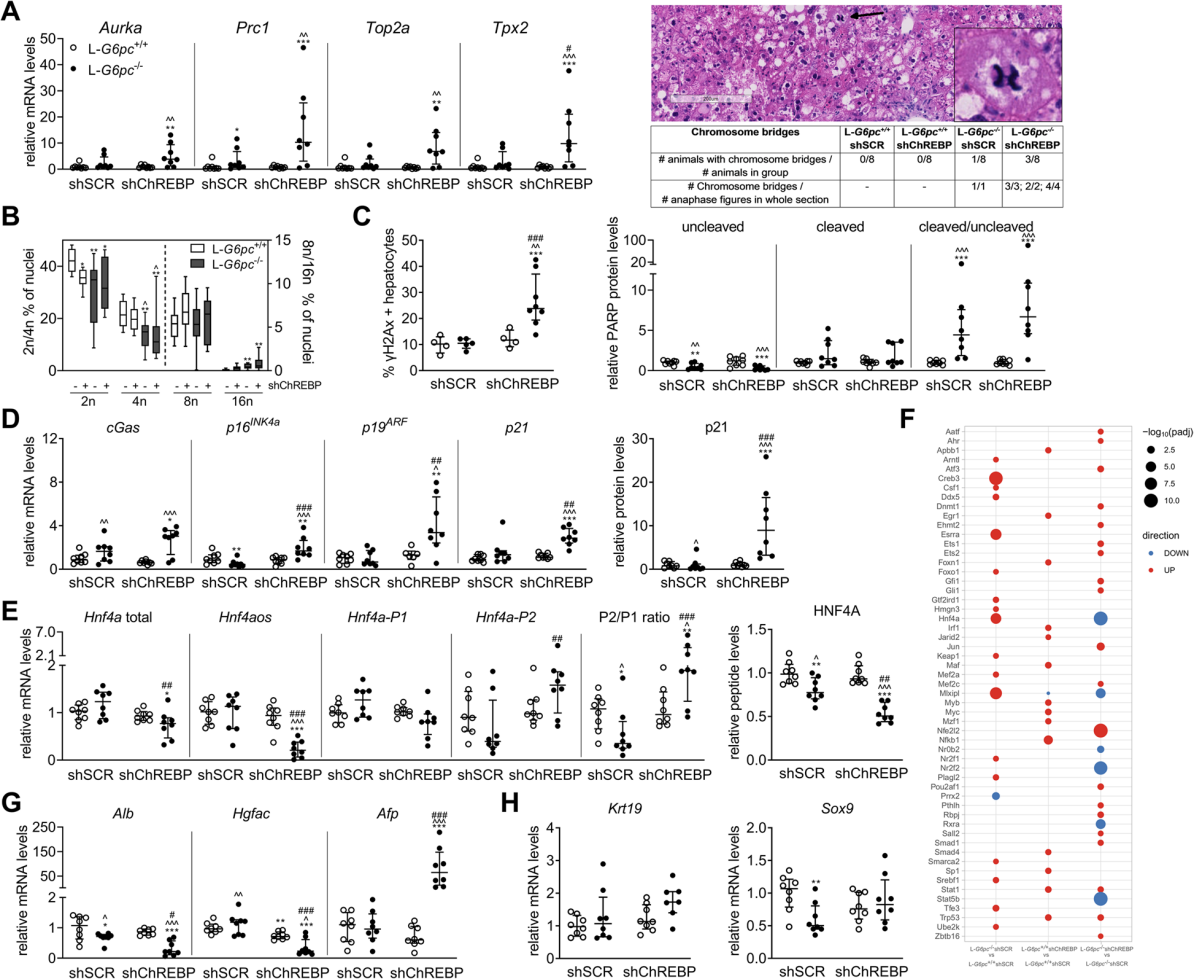
Prolonged ChREBP knockdown in L-*G6pc*^−/−^ mice induces DNA damage, cellular senescence, and hepatocyte dedifferentiation. Data after 21–26 days of shChREBP/L-*G6pc*^−/−^ and *n* = 8/group, unless stated otherwise. (**A**) CIN marker genes, spontaneous chromosome bridge incidence (with representative image), (**B**) Hepatocyte ploidy, (**C**) γH2Ax positivity (*n* = 4–8/group) and PARP protein expression, (**D**) *Cgas* and senescence-associated genes and p21 protein, and (**E**) HNF4A-related genes. (**F**) Reporter transcription factors analysis (after 10 days of shChREBP/L-*G6pc*^−/−^). (**G**-**H**) Hepatocyte differentiation marker genes (*n* = 7–8). Blots/Ponceau S: Fig. S4B-C. A/C-E/G-H: median ± interquartile range. B: box-and-whisker plots. A-E/G-H: Kruskal Wallis H-test, post-hoc Conover pairwise comparisons, *p < 0.05, **p < 0.01, ***p < 0.001 vs shSCR/L-*G6pc*^+/+^; ^ vs shChREBP/L-*G6pc*^+/+^; # vs shSCR/L-*G6pc*^−/−^

As short-term ChREBP-normalized hepatic GSD Ia mice suggested induction of chromosomal instability, DNA damage, cGAS-STING, and cellular senescence (Fig. 1F, Table 1), we also investigated these parameters upon prolonged ChREBP normalization. Prolonged ChREBP knockdown in L-*G6pc*^−/−^ mice induced chromosomal instability (CIN) marker genes (Fig. 4A). In parallel, histopathological analysis revealed an incidence of chromosome bridges, a hallmark of CIN [19], in these animals (Fig. 4A). Hepatic ChREBP knockdown also tended to further increase nuclear ploidy in L-*G6pc*^−/−^ mice (Fig. 4B). This was paralleled by a strong increase in γH2Ax positivity (Fig. 4C, Fig. S3A). PARP cleavage was not different between shChREBP- and shSCR/L-*G6pc*^−/−^ mice (Fig. 4C, Fig. S3B). shChREBP/L-*G6pc*^−/−^ mice showed increased mRNA levels of *Cgas* as well as the cellular senescence marker genes *p16*^*INK4a*^, *p19*^*ARF*^ (both encoded from the *Cdkn2a* locus), and *p21* (*Cdkn1a*), and a massive increase in hepatic p21 protein levels (Fig. 4D). Strikingly, hepatic ChREBP knockdown in L-*G6pc*^−/−^ mice reduced long non-coding *Hnf4aos* and total *Hnf4a* mRNA and HNF4A peptide levels, while increasing *Hnf4a-P2*/*Hnf4a-P1* ratio (Fig. 4E). As reduced *Hnf4aos* (HNF4A-AS1) and HNF4A expression are associated with hepatocyte dedifferentiation and advanced liver disease including liver cancer [20–27], these changes likely reflect hepatocellular dedifferentiation in shChREBP/L-*G6pc*^−/−^ mice. Reporter transcription factors analysis revealed that ChREBP- and HNF4A-targeted transcriptomes showed parallel responses to hepatic *G6pc* deficiency and combined *G6pc* deficiency/*Chrebp* normalization (Fig. 4F). In parallel, hepatic *Alb* and *Hgfac* mRNA levels were reduced and *Afp* expression was induced (Fig. 4G), while *Krt19* and *Sox9* remained unchanged (Fig. 4H). Taken together, these data indicate that prolonged ChREBP normalization in GSD Ia hepatocytes aggravates CIN while inducing DNA damage, cGAS-STING pathway activation, cellular senescence, and hepatocellular dedifferentiation.

## Discussion

The current study shows that normalization of hepatic ChREBP activity in GSD Ia liver induces progressive and extreme dysplastic liver growth, hepatocyte hypertrophy and -proliferation, YAP activation, cholestasis, CIN, DNA damage, cGAS-STING pathway activation, inflammation, cellular senescence, and hepatocellular dedifferentiation. Altogether, our data indicate that constitutive ChREBP activation in hepatic GSD Ia protects against advanced liver disease development, and disqualifies ChREBP as a therapeutic target for treatment of liver disease in GSD Ia.

A key finding in this study is that aggravation of hepatomegaly upon hepatic ChREBP knockdown in GSD Ia liver associates with enhanced nuclear levels and activity of YAP, a transcription factor that is critical for homeostatic control of liver size [28–32]. It was previously shown that hepatic YAP cooperates with ChREBP to regulate glycolytic and lipogenic gene expression [33], while our current work indicates that YAP is activated when ChREBP activity is reduced in hepatic GSD Ia. As we did not observe altered YAP activity upon hepatic ChREBP knockdown in wildtype mice, we propose that its activation is triggered by ChREBP-dependent physiological changes that occur within the context of hepatic GSD Ia. Among these, modulated bile acid metabolism was of primary interest to us, as we have previously implicated hepatic ChREBP in regulation of bile acid metabolism in GSD Ia [18], while hepatocyte YAP is activated upon high bile acid exposure [34, 35]. In agreement with these studies, plasma bile acids levels were increased upon hepatic ChREBP knockdown in GSD Ia liver. Moreover, the massive hepatocyte hypertrophy observed in ChREBP-normalized GSD Ia mice severely perturbed the cellular architecture of the liver, thereby likely distorting the bile canalicular system and impairing hepatic bile acid secretion. This may in turn have caused intrahepatic accumulation of bile acids and consequent YAP activation [35]. It was recently reported that accumulation of hepatic glycogen after 3 months of hepatocyte *G6pc* deletion induces hepatocyte phase separation and formation of glycogen-Mst1/2 aggregates. As this aggregation relieves the inhibitory phosphorylation of hepatic YAP by Mst1/2 signalling, it contributes to hepatomegaly in progressed GSD Ia [36]. Previous work [8, 37] and our current study indicate that attenuation of hepatic ChREBP activity aggravates hepatic glycogen storage in hepatic GSD Ia, while in the current study we show that ChREBP silencing activates hepatocyte YAP. However, as shSCR/L-*G6pc*^−/−^ mice did not exhibit hepatic YAP activation, and ChREBP normalization did not decrease YAP phosphorylation, glycogen-dependent Mst1/2 sequestration most likely contributes to YAP activation during advanced hepatic GSD Ia. As we primarily aimed to evaluate the role of ChREBP in liver disease progression in GSD Ia, the mechanisms underlying the observed YAP activation were not addressed and warrant follow-up studies.

An increased presence of chromosome bridges, induction of CIN marker genes, and enhanced DNA damage and hepatocyte death in shChREBP/L-*G6pc*^−/−^ mice indicate that ChREBP activation protects against chromosomal instability in hepatic GSD Ia. These changes likely reflect a high degree of hepatocellular stress and damage which may occur as a consequence of activated YAP [38]. On the other hand, DNA damage may trigger hepatocyte renewal through liver regeneration and YAP activation [39, 40]. However, the enrichment of CIN genes, enhanced PARP cleavage, and presence of chromosome bridges that occur in absence of YAP activation in shSCR/L-*G6pc*^−/−^ mice suggest that CIN/DNA damage occurs prior to YAP activation in early hepatic GSD Ia. Our data also indicate that ChREBP normalization in hepatic GSD Ia activates the cytosolic DNA-sensing cGAS-STING pathway [41, 42]. Enhanced cGAS-STING signalling, in turn, likely contributes to the observed induction of cellular senescence [41] in shChREBP/L-*G6pc*^−/−^ mice. Increased YAP activity, CIN, and aberrant cell division in ChREBP-normalized L-*G6pc*^−/−^ mice associated with increased hepatocyte dedifferentiation and trends towards increases in hepatocyte ploidy, in agreement with previous studies in non-GSD Ia contexts [29, 35, 37, 43–46]. Interestingly, the hepatic expression of *Hnf4aos,* a non-coding RNA which is associated with hepatocyte differentiation and, when decreased, has been linked to advanced liver disease in humans [25–27]*,* was lower in these animals. Consistently, ChREBP normalization in L-*G6pc*^−/−^ mice increased the ratio of the *Hnf4α* isoforms *Hnf4αP2/P1*, halved HNF4A protein expression levels, and suppressed HNF4α-regulated genes, which was consistently paralleled by induction of dedifferentiation- and proliferation-related gene expression [47].

Our finding that ChREBP controls the degree of hepatomegaly and the progression to non-alcoholic steatohepatitis (NASH) is in line with previous studies [37, 48, 49]. Importantly, our current work indicates that ChREBP activation in hepatic GSD Ia protects against hepatocellular dedifferentiation, and suggests that it may decelerate tumorigenesis. This is the first study that attributes a potential protective role for ChREBP in liver tumour development, and our findings are in line with published work showing that YAP expression induces or associates with liver tumour formation [28, 30, 34], that reduced HNF4A expression is linked to liver tumour risk in mice and humans [20–24], and that YAP represses HNF4A target genes [46]. The animal discomfort associated with extreme hepatomegaly that we observed upon attenuation of hepatic ChREBP activity in GSD Ia, however, prevented us from performing longer follow-up studies and thus assessment of liver tumour formation. Interestingly, although attenuation of ChREBP expression in GSD Ia mouse liver induced p53 activation and cell death, this was paralleled by increased proliferation, oncogenic YAP activation, and hepatocyte dedifferentiation. This is likely partly explained by the hepatic regenerative response induced in vivo, in which the consequence of hepatocyte death is not limited to single cells but impacts on the liver as a whole. Moreover, when comparing our current findings on hepatic GSD Ia to published work on hepatic ChREBP in liver tumour development [11, 15], its role appears to be disease-specific. Altogether, these insights underline the importance of establishing the context-specific roles of ChREBP to define its therapeutic potential for prevention and/or treatment of liver disease and tumour development.

## Conclusions

In summary, we show that ChREBP normalization in hepatic GSD Ia induces hepatocellular stress, chromosomal instability, DNA damage, and cGAS-STING pathway activation and provokes hepatocyte damage and inflammation, cellular senescence, and hepatocyte dedifferentiation. We hypothesize that hepatic YAP is induced to remove the damaged cells and to stimulate hepatocyte regeneration in order maintain liver function [40]. However, persistent metabolic stress, chromosomal instability, and DNA damage induced upon long-term ChREBP suppression in hepatic GSD Ia result in constitutive YAP activation, hence likely predisposing to liver tumorigenesis. Altogether, we propose that by sensing and balancing intracellular glucose levels [50], hepatic ChREBP decelerates hepatomegaly induction, liver disease progression, and hepatocellular tumour formation in GSD Ia.

## Financial support

This work was supported by a VIDI grant from the Dutch Scientific Organization, a grant from the Stichting Vrienden Beatrix Kinderziekenhuis (Foundation Friends Beatrix Children’s Hospital), and a grant from the De Cock-Hadders Foundation. In addition, this work is supported by European Union’s Horizon 2020 research and innovation program under the Marie Sklodowska-Curie grant agreement PoLiMeR, No 812616. M.H.O holds a Rosalind Franklin Fellowship from the University of Groningen.

## Supplementary Information

**Additional file 1:** Supplementary Materials and Methods, Supplementary Figures, SupplementaryTables, Supplementary References.

## Data Availability

RNA-sequencing data has previously been submitted to GEO (Gene Expression Omnibus) under GSE143357, which is yet to be made publicly available. Data generated or analysed during this study are included in this published article and its supplementary information files. Additional raw datasets and/or data files used and/or analysed during the current study are available from the corresponding author on reasonable request.

## Abbreviations

GSD Ia: Glycogen Storage Disease type 1a
G6PC/G6Pase-α: Glucose-6-phosphatase, catalytic subunit
G6P: Glucose-6-phosphate
ChREBPα/β (MLXIPL): Carbohydrate Response Element Binding Protein alpha/beta
L-*G6pc*^−^^/^^−^ mice: Hepatocyte-specific *G6pc* knockout mice
cGAS-STING: Cyclic GMP-AMP synthase-stimulator of interferon genes
IEM: Inborn error of metabolism
NAFLD: Non-alcoholic fatty liver disease
HCC: Hepatocellular carcinoma
dpt: Days post treatment (= days post final tamoxifen injection)
GSEA: Gene set enrichment analysis
IQR: Interquartile range
shRNA: Small hairpin RNA
siRNA: Small interfering RNA
YAP: Yes Associated Protein
CIN: Chromosomal instability
HNF4A: Hepatocyte Nuclear Factor 4 Alpha
*Hnf4aos*/HNF4A-AS1: HNF4A, opposite strand / HNF4A antisense RNA 1
*Alb*: Albumin
*Hgfac*: Hepatocyte growth factor activator
*Afp*: Alpha-fetoprotein
*Krt19*: Keratin 19
*Sox9*: SRY-box transcription factor 9
NASH: Non-alcoholic steatohepatitis
H&E: Hematoxylin&Eosin
PRCF: Percent relative cumulative frequency
IHC: Immunohistochemistry
