# Supplementary material for: Normalization of hepatic ChREBP activity does not protect against liver disease progression in a mouse model for Glycogen Storage Disease type Ia
Source: Cancer Metab. 2023 Apr 21;11:5. doi: 10.1186/s40170-023-00305-3 (PMC10122297; doi:10.1186/s40170-023-00305-3)
Supplement: Supplementary file 1 — Additional file 1: Supplementary Materials and Methods, Supplementary Figures, SupplementaryTables, Supplementary References. [file 40170_2023_305_MOESM1_ESM.docx]

**Supplementary material, accompanying the manuscript:**

Normalization of hepatic ChREBP activity does not protect against liver disease progression in a mouse model for Glycogen Storage Disease type Ia

**Authors**

Martijn G.S. Rutten^1^, Yu Lei^1^, Joanne H. Hoogerland^1^, Vincent W. Bloks^1^, Hong Yang^2^, Trijnie Bos^3^, Kishore A. Krishnamurthy^1^, Aycha Bleeker^1^, Mirjam H. Koster^1^, Rachel E. Thomas^4^, Justina C. Wolters^1^, Hilda van den Bos^5^, Gilles Mithieux^6,7,8^, Fabienne Rajas^6,7,8^, Adil Mardinoglu^2^, Diana C.J. Spierings^5^, Alain de Bruin^1,4^, Bart van de Sluis^1^, Maaike H. Oosterveer^1^

**Table of contents**

Supplementary Materials & Methods

Supplementary Figures

Supplementary Tables

Supplementary References

**Supplementary Materials & Methods**

*Construction, production and in vivo transduction of shRNAs using self-complementary AAV vectors*

To construct self-complementary AAV (scAAV) 2/8-U6-ChREBP, the scAAV2-LP1-hFIXco backbone vector [1] was restricted with BamHI and BbsI and the 3493 bp fragment was isolated and ligated. Restriction with BamHI removed hFIXco and partially deleted the LP1 promoter. The U6 promoter driving the expression of the construct was cloned into the vector in antisense orientation. shRNA construct directed against ChREBP (shChREBP) and scramble (shSCR) construct were ordered as oligonucleotides (shChREBP; 5’-aat tcA AAA AAT GTA GTT TGA AGA TGT GGG TCT CGA GAC CCA CAT CTT CAA ACT ACA TC-3’ and 3’-ggc caG ATG TAG TTT GAA GAT GTG GGT CTC GAG ACC CAC ATC TTC AAA CTA CAT TTT TT-5’, shSCR; 5’-aat tcG TTG TAA GTG GAG GTT TAA GTC TCG AGA CTT AAA CCT CCA CTT ACA ACA CCG GT-3’ and 3’-ggc caA CCG GTG TTG TAA GTG GAG GTT TAA GTC TCG AGA CTT AAA CCT CCA CTT ACA AC-5’), denatured at 99 °C for 10 min and annealed by cooling down to RT. The duplex oligonucleotides were cloned into the vector using EcoRI and AgeI. Production, purification and titration of the AAV2/8 viruses encoding the shRNA directed against ChREBP (AAV-shChREBP) and the scrambled control (AAV-shSCR) were performed as described [2,3].

*Animal experimentation details*

Animal age at shRNA infection was 13-18 weeks for the 10 days follow-up study; 9-14 weeks for the 20-21 days follow-up study; and 8-13 weeks for 25-26 days follow-up study. Sample size of each group is indicated in the legend of each figure. We performed a priori power analyses to compute the required sample size (using G*Power version 3.1.9), based on a α of 0.05 (two-tailed), power of 80%, and effect sizes based on expected means and standard deviations of parameters of primary interest. Animals were individually housed in individually ventilated cages (IVC) with wood bedding, nesting material and cardboard rolls in a light- and temperature-controlled facility (12h light / 12h dark cycle lighting regime) and fed a standard laboratory chow diet *ad libitum* (for 10-days follow-up study: RMH-B, Abdiets, Woerden, The Netherlands; for 20-21 days and 25-26 days follow-up studies: RM1, Special Diet Services, Essex, UK). In a separate cohort, animals were i.p. injected with BrdU (100 mg per kg body weight in PBS) at 8.30AM at 20-21 days after the last tamoxifen injection and were sacrificed 2 hours later. Animals were sacrificed by cardiac puncture under isoflurane anaesthesia and tissues were rapidly excised and stored. Hepatic water content was measured by weighing a piece of wet liver directly after excision, weighing its dry weight after evaporation of water at 70°C for 3-4 hours until stable, and calculating the relative decrease in weight. Correlation analysis on relative liver weight and total plasma bile acid levels was performed on two independent study cohorts on L-*G6pc*^+/+^ and L-*G6pc*^-/-^ mice treated with various doses of shSCR or shChREBP (64 mice in total).

*Histological and pathological analysis of the liver*

For microscopic examination, tissues were fixed in 4% (w/v) formaldehyde in PBS, embedded in paraffin, sectioned at 4 μm, and stained with Hematoxilin&Eosin (H&E). Stained sections were examined histologically for hepatocellular vacuolization, inflammatory foci, single cell death, and mitotic figures by a veterinary pathologist. Hepatic lipid accumulation was visualized by Oil red O staining of liver cryosections. Representative photomicrographs per liver were taken at 10x, 20x, or 40x using an DFC420 Camera (Leica, Wetzlar, Germany) or an Olympus DP26 Digital Camera (Olympus, Tokyo, Japan). Hepatocyte cell size was quantified manually for at least 120 hepatocytes per liver on H&E stained-microsopic images using imageJ (v1.50 and v1.53e, National Institutes of Health, Bethesda, MD). The spontaneous incidence of chromosome bridges was assessed by a veterinary pathologist.

Immunohistochemistry was carried out following an established protocol [4]. Briefly, paraffin-embedded tissue sections were deparaffinized in xylene and rehydrated in a graded series of alcohol. Antigen retrieval was performed using 10mM citrate buffer (pH 6.0) in a microwave (LG Intellowave, 15 minutes at 640 Watt) and the slides were left to cool to room temperature for 30 minutes. Endogenous peroxidase activity was blocked with 1% H_2_O_2_ in methanol for 30 min. The unspecific antigens were blocked in 10% normal goat serum for 30 minutes. Sections were incubated with primary antibodies rabbit anti Ki67 (RM-9106, Thermo, at 1:50 dilution), pH3 (06-570,Millipore,at 1:400 dilution), and γH2AX (9718S, Cell Signaling, at 1:500 dilution) and mouse anti BrdU (M0744, DAKO) at 4°C overnight and subsequently incubated with biotinylated goat-anti-rabbit secondary antibody (Vector Labs BA-1000 at 1:250) or horse-anti-mouse (Vector Labs BA-2000 at 1:125) for 30 min at room temperature. After 30 minutes incubation with Avidin-biotin ABC complex (PK-4000, Vector), the sections were washed and colorized with a 3,3′-diaminobenzidine (DAB) (Sigma, D5637) for 10 min and then counterstained with hematoxylin. Slides were scanned using a Nikon E800 microscope. Positive hepatocytes were counted by examination of at least three random high-power microscopic fields (magnification: 200x or 400x) in each section by a veterinary pathologist (for γH2AX, pH3 and KI67 in Fig. 1H-1I), counted in five random 20X microscopic fields by a veterinary pathologist (for BrdU in Fig. 3A), or counted in at least five random 20X microscopic fields by a researcher (for γH2AX in Fig. 4C). For pH3 and BrdU only strongly and homogenously stained nuclei were counted; for Ki67 and γH2AX a range of nuclear staining intensities from moderately stained stippled chromatin to intensely stained homogenous chromatin were considered positive. As significant differences in hepatocyte size were observed between groups (Figure 2C), which translate in a different number of hepatocytes per microscopic field, scores for Ki67-, pH3-, and γH2AX-positivity (Fig. 1G, 1I), as well as the incidence of mitotic figures, single cell death, and inflammatory foci (Fig. 1F, 1I, 2F, 3A), were corrected for the average hepatocyte size of each individual mouse. In order to do this, average hepatocyte sizes were manually quantified on H&E stained sections using Image J (Fig. 2C). Corrected scores for Ki67, pH3, γH2AX, mitotic figures, single cell death, and inflammatory foci were expressed relative to the average value in shSCR-treated L-*G6pc*^+/+^ animals. Where indicated, BrdU- and γH2AX-positivity was determined by dividing the number of positive hepatocytes in five random 20X fields by the total number of hepatocytes in five 20X fields (Fig. 3A, Fig. 4C).

*Biochemical assays*

Blood glucose was measured using an OneTouch Select Plus glucose meter (LifeScan, Inc., Milpitas, CA). Plasma ALT, lactate, ketone bodies, and free fatty acids were analysed using commercially available kits (Spinreact, Girona, Spain; Instruchemie, Delfzijl, The Netherlands; Wako, Neuss, Germany; Diasys, Holzheim, Germany). Plasma bile acid species were quantified using liquid chromatography-mass spectrometry [5]. Hepatic glycogen, glucose-6-phosphate (G6P), and phospholipid contents were quantified as described [6,7]. Hepatic lipids were extracted from 15% (w/v) liver homogenates in PBS according to Bligh & Dyer [8]. Hepatic TGs (Roche) and free (DiaSys) and total (Roche) cholesterol were analysed using commercially available kits. Hepatic cholesteryl-ester contents were calculated from the difference between total and free cholesterol contents.

*Gene expression analysis*

RNA was isolated using TRI-Reagent (Sigma-Aldrich). Complementary DNA (cDNA) was obtained by reverse transcription using M-MLV (Invitrogen) and amplified using primers and probes listed in Supplementary Table S1. mRNA levels were calculated based on a sample pool dilution curve and normalized to values in shSCR/L-*G6pc*^+/+^ mice. *36b4* (*Rplp0*) was selected as housekeeping gene for normalization.

*RNA-sequencing and gene set enrichment analysis (GSEA)*

For RNA sequencing, initial quality check and RNA quantification of the samples was performed by capillary electrophoresis using the LabChip GX (Perkin Elmer). Non-degraded RNA-samples were used for subsequent sequencing analysis. Sequence libraries were generated using the 3’QuantSeq sample preparation kits (LeXogen). The obtained cDNA fragment libraries were sequenced on an Illumina HiSeq2500 using default parameters (single read 1x50bp) in pools of multiple samples. The fastQ files where aligned to build Mus_musculus GRCm38 Ensemble Release 82 reference genome using HISAT [9] with default settings. Prior to gene quantification, SAMtools was used to sort the aligned reads [10]. The gene level quantification was performed by HTSeq-count [11]. The extracted raw count file (ENSG, counts) was analyzed using MADMAX [12]. After transformation (log_2_CPM), normalization (Voom) [13] was performed to generate the differentially expressed genes. Genome-wide expression analysis of the RNA-sequencing data (GSE143357, *to be made publicly available*) was performed using gene set enrichment analysis (GSEA) software version 2.2.4. The versions of the gene set database used were Mm_PathwaysOnly_GS.v3.gmt, a list of C2-pathway sets from KEGG, Biocarta, Reactome, and Wiki pathways, and a list of the hallmarks gene sets h.all.v6.1.symbols.gmt with addition of custom gene sets for chromosomal instability (CIN29; containing 29 genes related to CIN selected from the Carter70 set [14], cGAS-STING pathway (based on KEGG_CYTOSOLIC_DNA_SENSING_PATHWAY), and senescence (MMC2-senescence, a set of 241 genes based on Figure 3 of [15]). See supplementary table S2 for the corresponding lists of genes for all custom gene sets.

*Reporter transcription factors analysis*

To identify underlying transcriptional regulatory responses in shSCR- and shChREBP-treated L-*G6pc*^+/+^ and L-*G6pc*^-/-^ mice, we employed the Platform for Integrative Analysis of Omics (piano) R package [16] to perform so-called reporter transcription factors (TFs) analysis [17]. Differential expression of genes (log2-fold change) and corresponding significance levels (p-value) derived from the DESeq2 R package [18] in pairwise comparison, as well as a network of TFs and associated target genes from the TRRUST database [19], were used as input. The network contains 7,057 TF-target regulatory relationships of 827 mouse TFs collected based on text-mining following by manual curation. The reporter TFs in distinct-directional up class and in distinct-directional down class were chosen for presenting and were considered as significant when the corresponding adjusted p-value was less than 0.05 (padj < 0.05).

*Targeted proteomics*

Targeted proteomics was used to quantify G6PC, ChREBP, and HNF4A in delipidated homogenized liver tissue using isotopically labelled peptide standards as described previously [20]. Target peptides for ChREBP and HNF4A were (together with other peptides not discussed in this manuscript) concatenated into QconCATs, and additionally isotopically labelled peptide standard containing ^13^C-labeled lysine was added for G6PC (see Supplementary Table S3 for all peptides used). The peptide grade used was the PEPotec grade 2 from ThermoScientific. The concentrations of endogenous peptides were calculated from the known concentration of the standard, expressed in fmol/μg of total peptide, and subsequently expressed relative to the values in shSCR-treated L-*G6pc*^+/+^ animals.

*SDS-PAGE and Western blot*

To analyse protein expression in mouse liver in whole lysate, freeze-clamped liver was grinded to a powder. 10% (w/v%) homogenates of liver powder were prepared in RIPA buffer (50 mM Tris-HCl (pH 7.4), 150 mM NaCl, 1% Nonidet P-40, 1 mM PMSF, 2 mM EDTA, 1 mM sodium pyrophosphate (Na_4_P_2_O_7_), 50 mM sodium fluoride (NaF), 0.2 mM sodium orthovanadate (Na_3_VO_4_), and 1X Complete protease inhibitor cocktail (Roche Diagnostics, Mannheim, Germany)). The liver homogenates were homogenized using a TissueLyser (Qiagen) at 50 Hz for 2 min. Subsequently, the lysates were put on ice for at 30 min and vortexed every 10 min. The lysates were centrifuged at maximum speed for 10 min. at 4°C, and the supernatant was collected and stored at -80°C. Protein concentration was measured using the Bradford assay (#500-0006, Biorad) with a BSA standard (A2153, Sigma-Aldrich), and absorbance was measured at 595 nm on a Synergy H4 Hybrid platereader (Biotek). Equal amounts of lysate proteins (40 μg/well) were mixed with RIPA buffer and Laemmelli loading buffer (#161-0747, Biorad), boiled at 95°C for 5 min., and separated by 8-14% (where indicated) SDS-polyacrylamide gel electrophoresis. Proteins were transferred onto a Nitrocellulose membrane (Trans-Blot Turbo Midi Nitrocellulose Transfer Packs (170-4159, Biorad)) using the Trans-Blot Turbo Blotting System (Biorad) for detection of pYAP and YAP, or onto a polyvinylidene difluoride membrane (PVDF Blotting Membrane, A29574727, Amersham^TM^ Hybond^TM,^ GE Healthcare Life Science) using tankblotting for all other proteins, stained with Ponceau S to confirm equal loading (see Supplementary Figures), incubated in a blocking solution consisting of 5% BSA in TBST (10 mmol/L Tris–HCl (pH 8.0), 150 mmol/L NaCl, and 0.1% Tween 20) for 1h at RT, and incubated with primary antibody (1:1000) overnight at 4°C. Primary antibodies used were: rabbtit, anti-pYAP (YAP1 phospho S127, ab76252, Abcam), rabbit anti-YAP (4912, Cell Signaling), rabbit anti-PARP1 (9542, Cell Signaling), and rabbit anti-p21 (109199, Abcam). After overnight incubation with primary antibody, the membranes were washed with TBST for 3 times 10 min at room temperature before incubation with secondary antibodies (1:2000) for 1 hour at room temperature. Secondary antibodies used were: goat anti-rabbit IgG (P0448, Dako, for detection of YAP/pYAP), and goat anti-rabbit IgG (#1706515, Biorad, for detection of all other rabbit primary antibodies). Membranes were subsequently washed with TBST for 3 times 10 min at room temperature, and proteins were detected by chemiluminescence using the ChemiDoc Imaging System (Biorad, for detection of YAP/pYAP) or the ImageQuant LAS 4000 mini machine (Cytiva, GE Heathcare, for detection of all other proteins). Intensity of the bands was quantified using ImageQuant TL software (version 8.2.0, General Electric Company, for quantification of proteins detected on the ImageQuant), in which the rubber band method was used to substract background, or using Image Lab^TM^ software (version 6.0.0 build 12, Biorad, for quantification of proteins detected on the ChemiDoc). Bands were normalized for the average intensity of the bands of shSCR-treated L-*G6pc*^+/+^ mice. Ponceau S staining was used to confirm equal loading (see Supplementary Figures).

To analyse nuclear YAP protein expression, nuclear and cytosolic extracts of 20-25 freeze-clamped and crushed liver powder were prepared using NE-PER Nuclear and Cytoplasmic Extraction Reagents (78833/78835, ThermoScientific) according to manufacturer’s protocol (adaptation: liver powder was homogenized in appropriate volume of CER I using a Precellys Evolution bead-beating homogenizer (Bertin technologies) at 6000 Hz, 2 x 15 seconds with 30 seconds break). Protein concentration was measured using the BCA method using Pierce® BCA Protein Assay Kit (#23225, ThermoScientific) with a BSA standard (A2153, Sigma-Aldrich) according to the manufacturer’s protocol, and absorbance was measured at 540 nm on a Synergy H4 Hybrid platereader (Biotek). Equal amounts of lysate proteins (11 μg/well) were mixed with RIPA buffer and Laemmelli loading buffer (#161-0747, Biorad), boiled at 95°C for 5 min., and separated by 10% SDS-polyacrylamide gel electrophoresis. Procedures for blocking and antibody incubation steps, detection, and quantification are similar as for analysis of protein expression in whole lysate, as discussed above. Primary antibody used was rabbit anti-YAP (4912, Cell Signaling). Secondary antibody used was goat anti-rabbit IgG (#1706515, Biorad).

**Supplementary Figures**

**Fig. S1: Representative IHC images**

(A) Representative images (40X magnification) for γH2Ax, pH3, and Ki67 IHC (corresponding to data in Fig. 1G, 1I).

**Fig. S2: Validation of G6PC knockout / ChREBP knockdown, representative BrdU images, Western Blots, and Ponceau S stainings, and correlations between hepatic glycogen content and relative liver weight and relative *Ctgf* mRNA levels**

(A) Relative G6PC peptide levels, relative ChREBPα and –β mRNA and peptide levels, and relative mRNA levels of ChREBP-target *Pklr* in livers of mice after 21-26 days of combined G6PC knockout / ChREBP knockdown. N=7-8/group. (B) Representative BrdU images (20X magnification) of livers of mice after 20-21 days of combined G6PC knockout/ChREBP knockdown. (C) Representative Western Blot and Ponceau S staining as loading control for nuclear YAP protein expression in Figure 3C. (D) Representative Western Blot of pYAP and YAP protein levels used for calculation of pYAP/YAP ratio in Figure 3C, and quantification of relative pYAP protein levels. (E) Ponceau S staining as loading control for Western Blot on pYAP and YAP in whole liver lysate, presented in Figure 3C and Supplementary Figure S2D. Stainings are from the same samples that were run, in parallel, on different membranes (with different sample order). (F) Correlations between hepatic glycogen content and relative liver weight and relative hepatic *Ctgf* mRNA levels in shChREBP-treated L-*G6pc*^-/-^ mice. A, D: median ± interquartile range, Kruskal Wallis H-test, post-hoc Conover pairwise comparisons, *p < 0.05, **p < 0.01, ***p < 0.001 vs shSCR/L-*G6pc*^+/+^; ^ vs shChREBP/L-*G6pc*^+/+^; # vs shSCR/L-*G6pc*^-/-^.

**Fig. S3: Representative γH2Ax IHC images and Western Blots / Ponceau S stainings from Figure 4.**

(A) Representative histologic images (20X magnification) of γH2Ax staining in livers of mice after 21-26 days of combined G6PC knockout/ChREBP knockdown. (B-C) Representative Western Blot and Ponceau S staining as loading control for protein expression of (B) PARP in figure 4C and (C) p21 in Figure 4D.

**
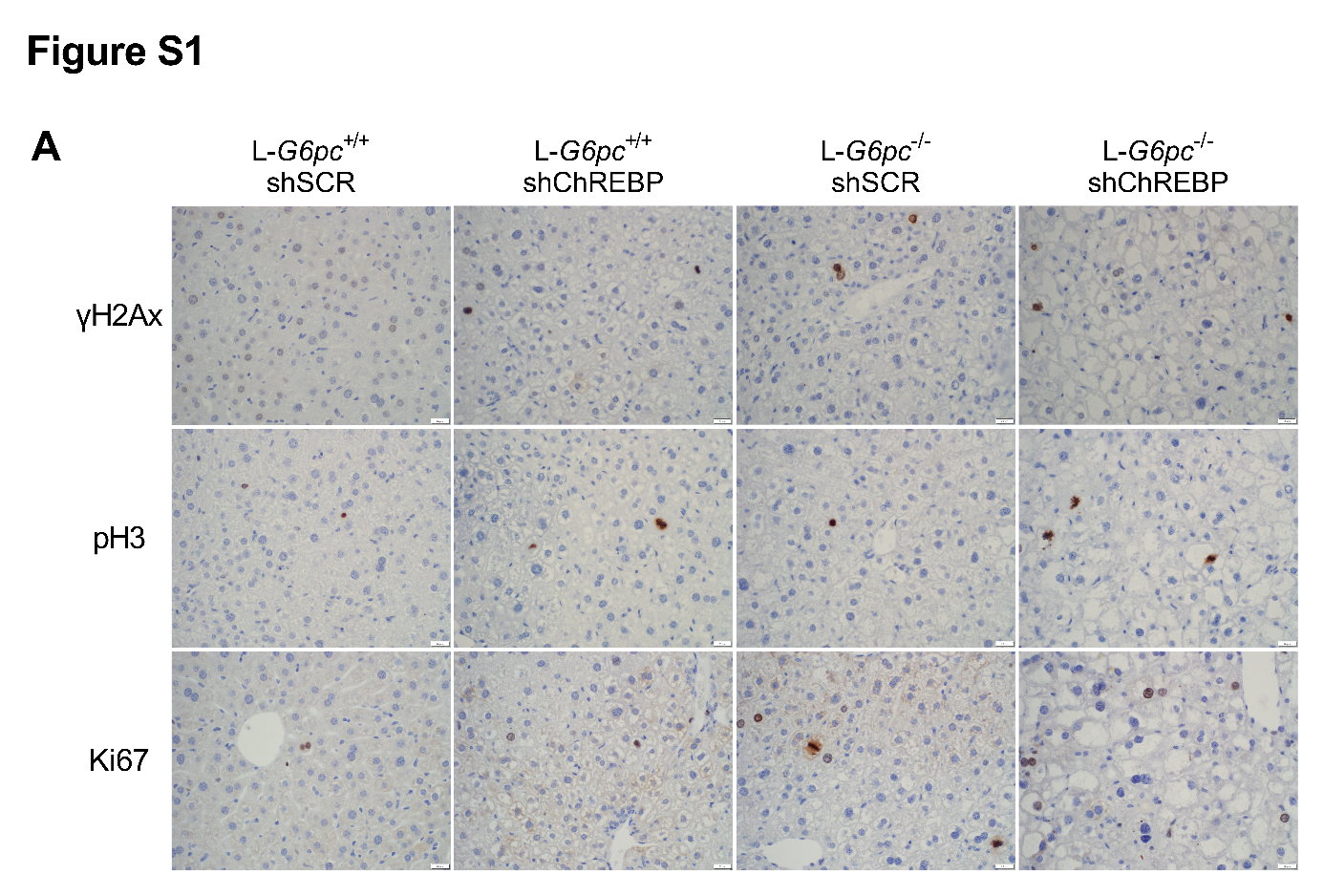
**

**
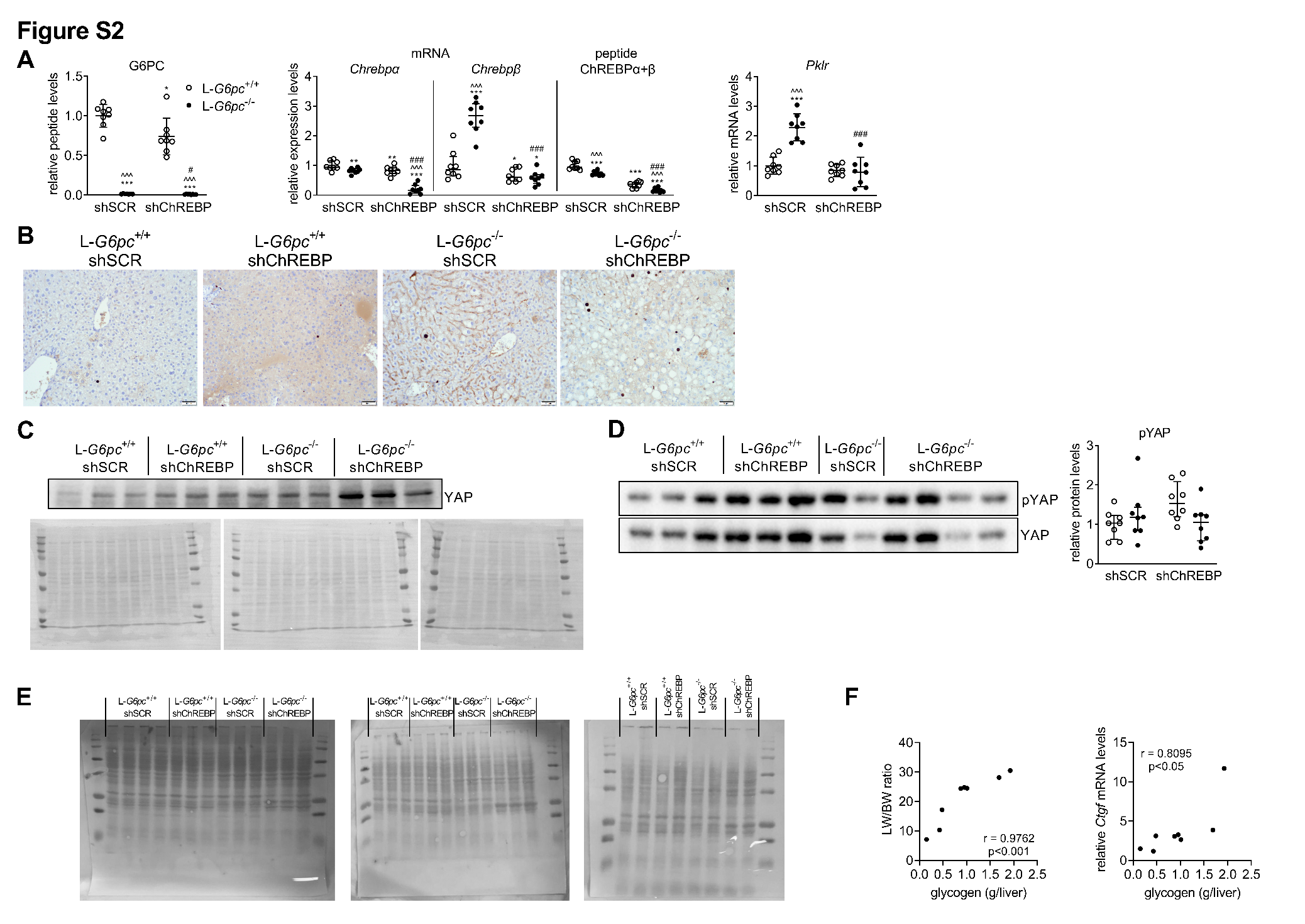
**

**
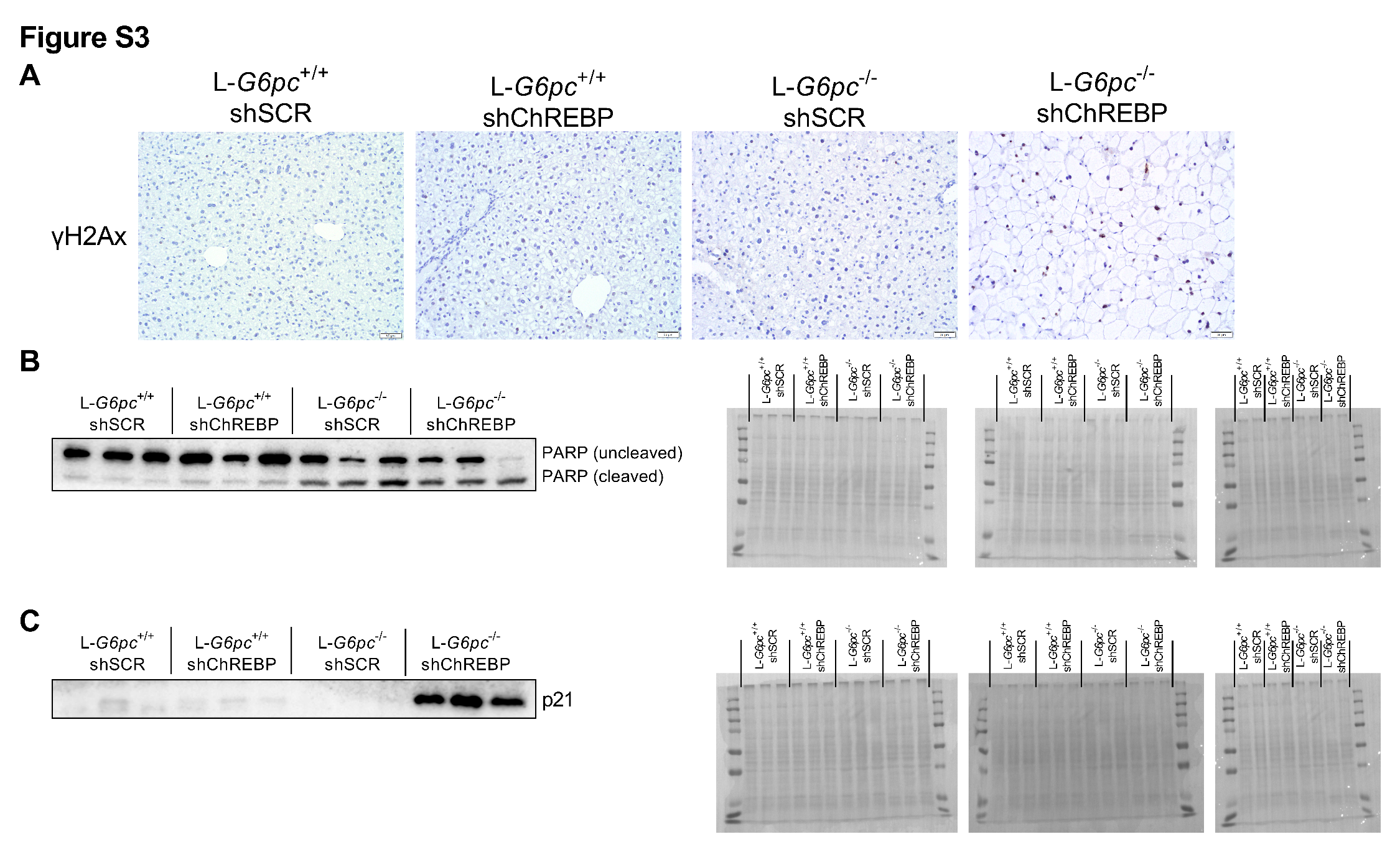
**

**Supplementary Tables**

**Table S1. Primers used for qPCR**

| Gene | Species | FW primers (5’ to 3’) | RV primer (5’ to 3’) | Probe (5’ to 3’) |
| --- | --- | --- | --- | --- |
| *36b4* (Taqman) | Mouse | GCT TCA TTG TGG GAG CAG ACA | CAT GGT GTT CTT GCC CAT CAG | TCC AAG CAG ATG CAG CAG ATC CGC |
| *36b4* (SYBR) | Mouse | GCT CCA AGC AGA TGC AGC A | CCG GAT GTG AGG CAG CAG |  |
| *Chrebpα* (*Mlxipl*) | Mouse | CGA CAC TCA CCC ACC TCT TC | TTG TTC AGC CGG ATC TTG TC | CCT GGC TTA CAG TGG CAA GCT GGT CTC T |
| *Chrebpβ*  (*Mlxipl*) | Mouse | TCT GCA GAT CGC GTG GAG | CTT GTC CCG GCA TAG CAA C | CTC AGT GGC AAG CTG GTC TCT CCC A |
| *Pklr* | Mouse | CGT TTG TGC CAC ACA GAT GCT | CAT TGG CCA CAT CGC TTG TCT | AGC ATG ATC ACT AAG GCT CGA CCA ACT CGG |
| *Cdkn1a (p21) (Fig. 1)* | Mouse | CGGTGTCAGAGTCTAGGGGA | AGGATTGGACATGGTGCCTG |  |
| *Il1β* (*Il1b*) | Mouse | ACC CTG CAG CTG GAG AGT GT | TTG ACT TCT ATC TTG TTG AAG ACA AAC C | CCC AAG CAA TAC CCA AAG AAG AAG ATG GAA |
| *Il6* | Mouse | CCG GAG AGG AGA CTT CAC AGA | AGA ATT GCC ATT GCA CAA CTC TT | ACC ACT TCA CAA GTC GGA GGC TTA ATT ACA |
| *Tnfα* (*Tnf*) | Mouse | GTA GCC CAC GTC GTA GCA AAC | AGT TGG TTG TCT TTG AGA TCC ATG | CGC TGG CTC AGC CAC TCC AGC |
| *Cd68* | Mouse | CAC TTC GGG CCA TGT TTC TC | AGG ACC AGG CCA ATG ATG AG | CAA CCG TGA CCA GTC CCT CTT GCT G |
| *Timp1* | Mouse | TCT GAG CCC TGC TCA GCA A | AAC AGG GAA ACA CTG TGC ACA C | CCA CAG CCA GCA CTA TAG GTC TTT GAG AAA GC |
| *Col1a1* | Mouse | CGG CTC CTG CTC CTC TTA GG | CTG ACT TCA GGG ATG TCT TCT TGG | CCA CTG CCC TCC TGA CGC ATG G |
| *αSMA* (*Acta2*) | Mouse | ACG AAC GCT TCC GCT GC | GAT GCC CGC TGA CTC CAT |  |
| *Mmp9* | Mouse | CCT GGA ACT CAC ACG ACA TCT TC | \| TGG AAA CTC ACA CGC CAG AA \| \| --- \| \|  \| |  |
| *Tgfβ* (*Tgfb1*) | Mouse | GCC CTT CCT GCT CCT CAT G | CCG CAC ACA GCA GTT CTT CTC |  |
| *Ctgf* (*Ccn2*) | Mouse | CCT GGT CCA GAC CAC AGA GT | TTT TCC TCC AGG TCA GCT TC |  |
| *Foxm1* | Mouse | CTG TGA GGG TCA AAG CTT GC | TCT GAT GTT TCA CTC GGG GC |  |
| *Spp1* | Mouse | TCA CCA TTC GGA TGA GTC TG | ACT TGT GGC TCT GAT GTT CC |  |
| *Birc5* | Mouse | ATC CAC TGC CCT ACC GAG AA | CTT GGC TCT CTG TCT GTC CAG TT |  |
| *Ccnd1* | Mouse | TCA AGT GTG ACC CGG ACT GC | CCT TGG GGT CGA CGT TCT G |  |
| *Cyr61* | Mouse | AGA GGC TTC CTG TCT TTG GC | CCA AGA CGT GGT CTG AAC GA |  |
| *Areg* | Mouse | TTG CTG CTG GTC TTA GGC TC | TCT GAG TAG TCA TAG TCG GCT C |  |
| *Itgb2* | Mouse | GCT TTG GGT CGT TTG TGG AC | TGC CGA CCT CTG TCT GAA AC |  |
| *Shp* | Mouse | AAG GGC ACG ATC CTC TTC AA | CTG TTG CAG GTG TGC GAT GT | ATG TGC CAG GCC TCC GTG CC |
| *Cyp7a1* | Mouse | CAG GGA GAT GCT CTG TGT TCA | AGG CAT ACA TCC CTT CCG TGA | TGC AAA ACC TCC AAT CTG TCA TGA GAC CTC C |
| *Cyp7b1* | Mouse | TGA AAT AGG AGC ACA TCA TCT TGG | AAT ACA TTG CCC AGA ACA TAG CTG |  |
| *Cyp8b1* | Mouse | AAG GCT GGC TTC CTG AGC TT | AAC AGC TCA TCG GCC TCA TC |  |
| *Cyp27a1* | Mouse | GCC TTG CAC AAG GAA GTG ACT | CGC AGG GTC TCC TTA ATC ACA |  |
| *Ntcp* (*Slc10a1*) | Mouse | TCCGTCGTAGATTCCTTTGC | AGGGGGACATGAACCTCAG |  |
| *Bsep* (*Abcb11*) | Mouse | GACTTTCCACAGTGGCGTCT | TCACTCAACAACCCTACAGATG |  |
| *Aurka* | Mouse | CTGGATGCTGCAAACGGATAG | CGCTGGGAGTTAGAAGGACAC |  |
| *Prc1* | Mouse | AAGGAGCTGAGTACCCTGTG | CAGAGGATGTCACGGAGTTCT |  |
| *Top2a* | Mouse | AACAAAGGGACCCAAAAATGTCT | TGTGTTCAACAACAGGGATTCC |  |
| *Tpx2* | Mouse | CACACCGTTGAAGGCAGTTG | ACAGCTCTCTTAGCATCCAGG |  |
| *Cgas* | Mouse | GTCGGAGTTCAAAGGTGTGGA | GACTCAGCGGATTTCCTCGTG |  |
| *p16^INK4a^* (*Cdkn2a* locus) | Mouse | AATCTCCGCGAGGAAAGC | GTCTGCAGCGGACTCCAT |  |
| *p19^ARF^* (*Cdkn2a* locus) | Mouse | CATGTTGTTGAGGCTAGAGAG | GAATCTGCACCGTAGTTGAG |  |
| *Cdkn1a (p21) (Fig. 4)* | Mouse | TTG TCG CTG TCT TGC ACT CTG | GCT TGG AGT GAT AGA AAT CTG TCA G | CTG CCT CCG TTT TCG GCC CTG |
| *Hnf4a* total | Mouse | AAA CAC TAC GGA GCC TCG AG | TCT ACC ACA CAT TGT CGG CT |  |
| *Hnf4aos* | Mouse | GAG CAC GTG TGT CCA TTT GG | GCC TTC ATT TCT TGT CT GCG |  |
| *Hnf4a-P1* | Mouse | GGT AGG GGA GAA TGC GAC TC | CAC CTT CAG ATG GGG ACG TG |  |
| *Hnf4a-P2* | Mouse | ACC CTT GGT CAT GGT CAG TG | CAC CTT CAG ATG GGG ACG TG |  |
| *Albumin* (*Alb*) | Mouse | TGC TTT TTC CAG GGG TGT GTT | TTA CTT CCT GCA CTA ATT TGG CA |  |
| *Hgfac* | Mouse | AAG TGC TCC AGC AGC AGA GAC | CTC AGA GGT ACA GGA GTG CAG C |  |
| *Alpha-fetoprotein* (*Afp*) | Mouse | CTT CCC TCA TCC TCC TGC TAC | ACA AAC TGG GTA AAG GTG ATG G |  |
| *Krt19* | Mouse | GTC CTA CAG ATT GAC AAT GC | CAC GCT CTG GAT CTG TGA CA |  |
| *Sox9* | Mouse | GAG CCG GAT CTG AAG AGG GA | GCT TGA CGT GTG GCT TGT TC |  |

**Table S2. Composition of custom gene sets added to GSEA (Table 1)**

| **CIN29** |
| --- |
| AURKA |
| AURKB |
| CCNB1 |
| CCNB2 |
| CDC20 |
| CDCA8 |
| CDK1 |
| CEP250 |
| CEP55 |
| CKAP5 |
| ECT2 |
| ESPL1 |
| FOXM1 |
| H2AFX |
| KIF20A |
| KIF4 |
| MAD2L1 |
| NCAPG2 |
| NCAPH |
| NEK2 |
| PRC1 |
| PTTG1 |
| RAD21 |
| RFC4 |
| TOP2A |
| TPX2 |
| TTK |
| ZWILCH |
| ZWINT |

| **MMC2-senescence** | | | | | |
| --- | --- | --- | --- | --- | --- |
| BNIP5 | ARL14 | DUSP10 | KIF3C | DTYMK | HMGB1P5 |
| XIRP1 | H2AC6 | CTSF | HLA-E | EXOSC8 | MCM5 |
| MMP3 | CCN3 | HLA-J | HAUS7 | HAUS1 | CDCA7 |
| FOLR2 | CDKN1A | LACC1 | FANCE | ACAT2 | CDT1 |
| LINC00520 | AA467197 | PSMG3-AS1 | SLC19A1 | DUT | ATAD5 |
| HSD3BP5 | ADGRF4 | CPEB2 | RTEL1 | HNRNPA1P48 | FANCD2 |
| UBD | H2BC5 | ZNFX1 | RPA3 | SNRPA | TMPO |
| ACTBL2 | QPCT | ITGB8 | RANBP1 | HIRIP3 | PTTG1 |
| CST1 | H2BC4 | CALCOCO1 | PFAS | RTEL1-TNFRSF6B | UHRF1 |
| CSF2 | BIRC3 | LAMB3 | SNHG3 | NDC1 | DLEU2 |
| RAI2 | IRF9 | HLA-C | H1F10 | SAPCD2 | POLE2 |
| GIMAP6 | H2BC18 | TMOD2 | ALYREF | DYNC2I2 | PRIM1 |
| CXCL11 | ITGB6 | AMPD3 | HNRNPA2B1 | CENPV | DEPDC1B |
| KRT17 | TMEM217 | PPP2R5B | HAUS5 | NUP210 | SPC24 |
| IGFL2 | PLB1 | SP100 | CCDC138 | H2AX | HMGB2 |
| SLC6A3 | DDX58 | CARD6 | MIS18A | CKS1B | LMNB1 |
| LUM | HLA-B | PBXIP1 | NCAPD3 | POLD1 |  |
| IL6 | TP53INP1 | TAP1 | COQ3 | MMS22L |  |
| NEURL3 | FBXO32 | YPEL5 | HAUS4 | TUBA1B |  |
| CXCL10 | GBP3 | ZFYVE1 | USP1 | FBL |  |
| VNN1 | FILIP1L | C8orf31 | TEDC1 | CHAF1B |  |
| SERPINB7 | TMEM86A | APOL2 | DEK | LBR |  |
| CHI3L2 | H3C6 | GRN | POLD3 | FANCG |  |
| TMEM140 | BTG2 | LINC00886 | CDCA4 | PSIP1 |  |
| DUOX2 | LAMC2 | GADD45A | SUV39H1 | RPL12P38 |  |
| PLA2G4C | YPEL2 | RELB | CCDC34 | TIMELESS |  |
| H4C8 | H3C10 | IRAK2 | TFAP4 | UBE2T |  |
| RTL9 | ARHGEF37 | TAPBPL | DNMT1 | ARHGAP19 |  |
| NXF3 | POPDC2 | SUSD6 | PTMA | RFC4 |  |
| MAF | OPTN | FAM214A | NSD2 | ANP32E |  |
| ACE2 | ATP6V1FNB | B2M | POLE | NUDT1 |  |
| SPATA18 | SLFN5 | ERAP2 | HNRNPA1 | MCM6 |  |
| ABCA12 | TNFAIP3 | NEU1 | ANP32B | RFC5 |  |
| TNFSF15 | DNAI4 | PNMA2 | TONSL | KIF22 |  |
| COL17A1 | AOX1 | FAM102A | RNASEH2A | HMGN2 |  |
| ITGAM | FAM214B | FBXL2 | CENPJ | H2AZ1 |  |
| H2BC21 | MR1 | DNAJB2 | INCENP | DNA2 |  |
| TG | GABARAPL1 | TIMP2 | TAF9B | ITGB3BP |  |
| CTSS | CREBRF | PSAP | SKP2 | KNTC1 |  |
| NECTIN4 | PGM2L1 | IRF1 | DBF4 | MCM7 |  |
| PIK3IP1 | NATD1 | SRPX2 | SSRP1 | MCM2 |  |
| GPNMB | H2BC15 | CYLD | HNRNPD | PSRC1 |  |
| TFEC | AQP7 | PINK1 | PHF19 | NRM |  |
| DAPP1 | RUNDC3A | ARMCX3 | IMPA2 | FSBP |  |
| NCF2 | H2BC6 | MAPK11 | NASP | HMGB1 |  |

**Table S3. Peptides used for targeted proteomics.**

| **Protein target** | **Peptide** |
| --- | --- |
| G6PC | GLGVDLLWTLEK |
| MLXIPL (ChREBP) | LGFDTLHGLVSTLSAQPSLK |
| HNF4A | AIIFFDPDAK |

**Supplementary References**

[1] Nathwani AC, Gray JT, Ng CYC, Zhou J, Spence Y, Waddington SN, et al. Self-complementary adeno-associated virus vectors containing a novel liver-specific human factor IX expression cassette enable highly efficient transduction of murine and nonhuman primate liver. Blood 2006;107:2653–61. https://doi.org/10.1182/blood-2005-10-4035.

[2] Hermens WTJMC, Brake O Ter, Dijkhuizen PA, Sonnemans MAF, Grimm D, Kleinschmidt JA, et al. Purification of recombinant adeno-associated virus by iodixanol gradient ultracentrifugation allows rapid and reproducible preparation of vector stocks for gene transfer in the nervous system. Hum Gene Ther 1999;10:1885–91. https://doi.org/10.1089/10430349950017563.

[3] Seppen J, Bakker C, de Jong B, Kunne C, van den Oever K, Vandenberghe K, et al. Adeno-associated Virus Vector Serotypes Mediate Sustained Correction of Bilirubin UDP Glucuronosyltransferase Deficiency in Rats. Mol Ther 2006;13:1085–92. https://doi.org/10.1016/j.ymthe.2006.01.014.

[4] Ptak A, Rak-Mardyła A, Gregoraszczuk EL. Cooperation of bisphenol A and leptin in inhibition of caspase-3 expression and activity in OVCAR-3 ovarian cancer cells. Toxicol Vitr 2013;27:1937–43. https://doi.org/10.1016/j.tiv.2013.06.017.

[5] de Boer JF, Schonewille M, Boesjes M, Wolters H, Bloks VW, Bos T, et al. Intestinal Farnesoid X Receptor Controls Transintestinal Cholesterol Excretion in Mice. Gastroenterology 2017;152:1126-1138.e6. https://doi.org/10.1053/j.gastro.2016.12.037.

[6] Bergmeyer HU, Gawehn K. Principles of enzymatic analysis. Verlag Chemie; 1978.

[7] Böttcher CJF, gent CM Van, Pries C. A rapid and sensitive sub-micro phosphorus determination. Anal Chim Acta 1961;24:203–4. https://doi.org/10.1016/0003-2670(61)80041-x.

[8] Bligh EG, Dyer WJ. A rapid method of total lipid extraction and purification. Can J Biochem Physiol 1959;37:911–7. https://doi.org/10.1139/o59-099.

[9] Kim D, Langmead B, Salzberg SL. HISAT: A fast spliced aligner with low memory requirements. Nat Methods 2015;12:357–60. https://doi.org/10.1038/nmeth.3317.

[10] Li H, Handsaker B, Wysoker A, Fennell T, Ruan J, Homer N, et al. The Sequence Alignment/Map format and SAMtools. Bioinformatics 2009;25:2078–9. https://doi.org/10.1093/bioinformatics/btp352.

[11] Anders S, Pyl PT, Huber W. HTSeq-A Python framework to work with high-throughput sequencing data. Bioinformatics 2015;31:166–9. https://doi.org/10.1093/bioinformatics/btu638.

[12] Lin K, Kools H, de Groot PJ, Gavai AK, Basnet RK, Cheng F, et al. MADMAX - Management and analysis database for multiple ~omics experiments. J Integr Bioinform 2011;8:160. https://doi.org/10.1515/jib-2011-160.

[13] Law CW, Chen Y, Shi W, Smyth GK. Voom: Precision weights unlock linear model analysis tools for RNA-seq read counts. Genome Biol 2014;15. https://doi.org/10.1186/gb-2014-15-2-r29.

[14] Carter SL, Eklund AC, Kohane IS, Harris LN, Szallasi Z. A signature of chromosomal instability inferred from gene expression profiles predicts clinical outcome in multiple human cancers. Nat Genet 2006;38:1043–8. https://doi.org/10.1038/ng1861.

[15] Jochems F, Thijssen B, De Conti G, Jansen R, Pogacar Z, Groot K, et al. The Cancer SENESCopedia: A delineation of cancer cell senescence. Cell Rep 2021;36. https://doi.org/10.1016/j.celrep.2021.109441.

[16] Väremo L, Nielsen J, Nookaew I. Enriching the gene set analysis of genome-wide data by incorporating directionality of gene expression and combining statistical hypotheses and methods. Nucleic Acids Res 2013;41:4378–91. https://doi.org/10.1093/nar/gkt111.

[17] Oliveira AP, Patil KR, Nielsen J. Architecture of transcriptional regulatory circuits is knitted over the topology of bio-molecular interaction networks. BMC Syst Biol 2008;2. https://doi.org/10.1186/1752-0509-2-17.

[18] Love MI, Huber W, Anders S. Moderated estimation of fold change and dispersion for RNA-seq data with DESeq2. Genome Biol 2014;15. https://doi.org/10.1186/s13059-014-0550-8.

[19] Han H, Cho JW, Lee S, Yun A, Kim H, Bae D, et al. TRRUST v2: An expanded reference database of human and mouse transcriptional regulatory interactions. Nucleic Acids Res 2018;46:D380–6. https://doi.org/10.1093/nar/gkx1013.

[20] Hoogerland JA, Lei Y, Wolters JC, de Boer JF, Bos T, Bleeker A, et al. Glucose‐6‐phosphate regulates hepatic bile acid synthesis in mice. Hepatology 2019;0:1–14. https://doi.org/10.1002/hep.30778.
